# Supplementary material for: Predicting visual function by interpreting a neuronal wiring diagram
Source: Nature. 2024 Oct 2;634(8032):113–23. doi: 10.1038/s41586-024-07953-5 (PMC11446822; doi:10.1038/s41586-024-07953-5)
Supplement: Supplementary file 6 — CRF and ERF predictions for individual TmY4 and TmY9 cells. Analogous to Supplementary Data 3, but for TmY target types. Shown are the top four monosynaptic pathways, the strongest pathway passing through each of the top ten intermediary types (ranking from Extended Data Fig. 7), and the trisynaptic pathway Tm1–TmY–Dm3–TmY (see the section entitled Prediction of spatial normalization). [file 41586_2024_7953_MOESM6_ESM.zip › DataS4/TmY9qΓèÑ/720575940635611802.pdf]

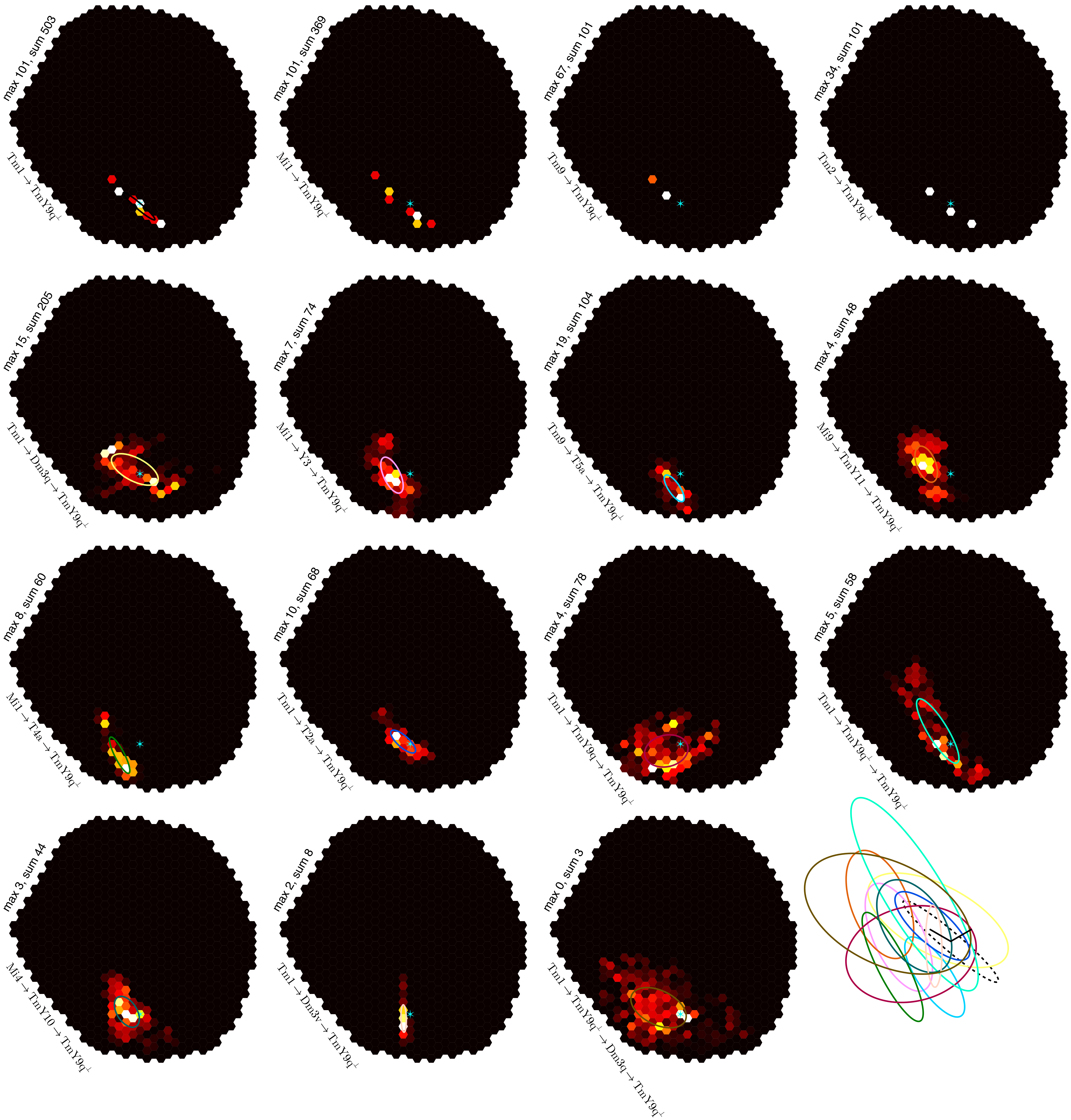
max 101, sum 503
Tm1 → TmY9q⊥
max 101, sum 369
Mi1 → TmY9q⊥
max 67, sum 101
Tm9 → TmY9q⊥
max 34, sum 101
Tm2 → TmY9q⊥
max 15, sum 205
Tm1 → Dm3q → TmY9q⊥
max 7, sum 74
Mi1 → Y3 → TmY9q⊥
max 19, sum 104
Tm9 → T5a → TmY9q⊥
max 4, sum 48
Mi9 → TmY11 → TmY9q⊥
max 8, sum 60
Mi1 → T4a → TmY9q⊥
max 10, sum 68
Tm1 → T2a → TmY9q⊥
max 4, sum 78
Tm1 → TmY9q → TmY9q⊥
max 5, sum 58
Tm1 → TmY9q⊥ → TmY9q⊥
max 3, sum 44
Mi4 → TmY10 → TmY9q⊥
max 2, sum 8
Tm1 → Dm3v → TmY9q⊥
max 0, sum 3
Tm1 → TmY9q⊥ → Dm3q → TmY9q⊥
